# O Índice de Imuno Inflamação Sistêmica Prevê Mortalidade Hospitalar em Pacientes Submetidos à Cirurgia Cardíaca com Circulação Extracorpórea

**DOI:** 10.36660/abc.20230245

**Published:** 2024-03-28

**Authors:** İnayet Güntürk, Rifat Ozmen, Okan Ozocak, Ertuğrul Emre Güntürk, Fatma Dagli, Cevat Yazici

**Affiliations:** 1 Nigde Omer Halisdemir University Faculty of Health Sciences Nigde Turquia Nigde Omer Halisdemir University, Faculty of Health Sciences, Midwifery, Nigde – Turquia; 2 Erciyes University Faculty of Medicine Kayseri Turquia Erciyes University, Faculty of Medicine, Cardiovasculary Surgery, Kayseri – Turquia; 3 Acibadem Hospitals Group Istanbul Turquia Acibadem Hospitals Group – Cardiology, Istanbul – Turquia; 4 Cetin Sen Science and Art Center Kayseri Turquia Cetin Sen Science and Art Center – Chemistry, Kayseri – Turquia; 5 Erciyes University Faculty of Medicine Kayseri Turquia Erciyes University Faculty of Medicine, Clinical Biochemistry, Kayseri – Turquia

**Keywords:** Circulação Extracorpórea, Inflamação, Mortalidade Hospitalar, Cirurgia Torácica

## Abstract

**Fundamento:**

O índice de imuno-inflamação sistêmica (SII), um novo índice inflamatório calculado usando contagens de plaquetas, neutrófilos e linfócitos, demonstrou ser um fator de risco independente para a identificação de doença arterial coronariana de alto risco em pacientes submetidos a intervenção coronária percutânea e cardiovascular e cirurgia com circulação extracorpórea (CEC). A relação entre as taxas de mortalidade relacionadas ao SII e à CEC permanece obscura.

**Objetivo:**

Esta pesquisa foi desenhada para investigar o uso do SII para prever mortalidade hospitalar em pacientes submetidos à cirurgia cardíaca com CEC.

**Métodos:**

Quatrocentos e oitenta pacientes submetidos a procedimento cardíaco envolvendo CEC durante 3 anos foram coletados do banco de dados do hospital. Foram comparados os dados demográficos, comorbidades, perfis hematológicos e bioquímico e dados operatórios dos grupos. Análises múltiplas de regressão logística foram feitas para determinar preditores independentes de mortalidade. Os fatores prognósticos foram avaliados por análise multivariada e os valores preditivos de SII, relação neutrófilo-linfócito (NLR) e razão plaqueta-linfócito (PLR) para mortalidade foram comparados. Um valor de p <0,05 foi considerado significativo.

**Resultados:**

Dos 480 pacientes, 78 desenvolveram mortalidade hospitalar após cirurgia cardíaca. O SII foi um preditor independente de mortalidade hospitalar (odds ratio: 1,003, intervalo de confiança de 95%: 1,001-1,005, p<0,001). O valor de corte do SII foi >811,93 com sensibilidade de 65% e especificidade de 65% (área sob a curva: 0,690). Os valores preditivos de SII, PLR e NLR foram próximos entre si.

**Conclusão:**

Altos escores pré-operatórios do SII podem ser usados para determinação precoce de tratamentos apropriados, o que pode melhorar os resultados cirúrgicos de cirurgia cardíaca no futuro.

**Figure:**
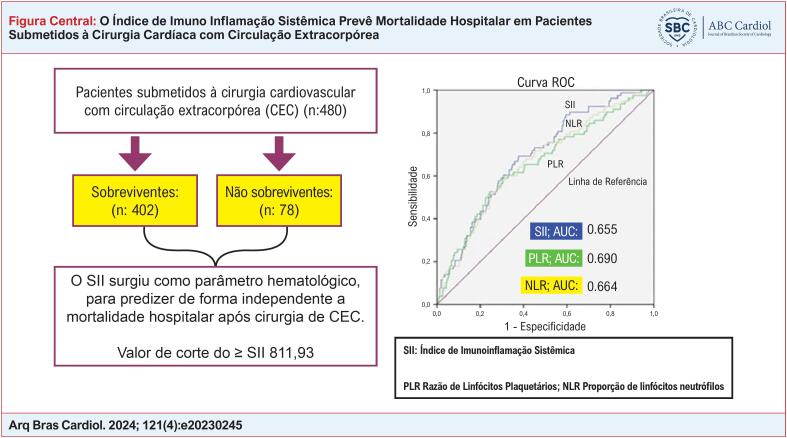

## Introdução

As técnicas de circulação extracorpórea (CEC), especialmente a CEC durante a cirurgia cardíaca, fornecem fluxo sanguíneo e oxigênio aos tecidos e órgãos.^1^ A CEC tornou-se o padrão para muitos procedimentos cardíacos e seu avanço está possibilitando avanços na cirurgia cardiovascular. O procedimento é considerado relativamente seguro.^2^ A CEC, por outro lado, é bem conhecida por iniciar uma cascata de reações inflamatórias, e essa resposta inflamatória tem sérias consequências clínicas.^2 - 4^ Como resultado, investigar o papel das alterações inflamatórias no prognóstico do paciente tornou-se um alvo de alta prioridade para o desenvolvimento de estratégias terapêuticas e preventivas.^5^ Para esse fim, muitos mediadores, como níveis de citocinas pró-inflamatórias, como IL-6, TNF, sistema de coagulação/fibrinolítico e marcadores de ativação do complemento foram investigados em relação à resposta inflamatória relacionada à CEC.^5 , 6^

Porém, quando são considerados fatores como facilidade de mensuração e interpretação, bem como baixo custo, tais parâmetros de hemograma continuam a ser amplamente utilizados. Embora um aumento no número de glóbulos brancos seja aceito como um marcador geral para a resposta inflamatória desencadeada por vários estimulantes, seu valor preditivo é insuficiente.^7^ Portanto, há uma ênfase recente em índices hematológicos, como a relação neutrófilos-linfócitos (RNL) e relação plaquetas-linfócitos (RPL), como marcadores de inflamação perioperatória, considerando a proposição de complicações pós-operatórias.^8^

O índice de inflamação imunológica sistêmica (SII), um novo índice inflamatório calculado usando contagens de plaquetas, neutrófilos e linfócitos, demonstrou ser um forte marcador prognóstico em uma variedade de cânceres.^9 , 10^ Além disso, foi demonstrado que o SII é fator de risco independente para identificação de doença arterial coronariana de alto risco em pacientes submetidos à intervenção coronariana percutânea, fibrilação atrial pós-operatória após cirurgia de revascularização do miocárdio, fator prognóstico para endocardite infecciosa e presença de ectasia arterial coronariana isolada.^11 - 14^ Porém, a relação entre SII e complicações relacionadas à CEC, especialmente em termos de taxa de mortalidade hospitalar, permanece obscura.

Diante disso, o presente estudo procurou elucidar a associação independente entre SII e a ocorrência de mortalidade em curto prazo em pacientes submetidos à cirurgia cardíaca com CEC, a fim de fornecer um marcador preditivo para intervenções terapêuticas o mais precocemente possível.

## Métodos

O estudo foi realizado e aprovado pelo Comitê de Ética Local (Número de aprovação: 2021/608). Devido à natureza retrospectiva do estudo, o consentimento informado individual por escrito foi dispensado.

Foram coletados pacientes submetidos a procedimento cardíaco envolvendo CEC ao longo de 3 anos (de 2018 a 2021). Não houve seleção baseada na operação usada. Dados pré-operatórios de 480 pacientes adultos foram obtidos do banco de dados do hospital. Os seguintes dados foram coletados para todos os sujeitos: dados demográficos (idade, sexo, tabagismo); presença de comorbidades (doença pulmonar obstrutiva crônica, hipertensão arterial, dislipidemia, diabetes medicamentoso e insuficiência renal crônica); resultados laboratoriais pré-operatórios [hemograma completo (CBC), nitrogênio ureico no sangue (BUN), creatinina sérica (SCr), AST, ALT, colesterol total (C-Total), HDL-C, LDL-C, glucose,… etc.]; tipo de operação cirúrgica, CEC e duração do pinçamento aórtico. O SII foi definido como contagem de plaquetas x contagem de neutrófilos/linfócitos. Foram excluídos pacientes submetidos a suporte de vida extracorpóreo pré-operatório, infecção ativa, condições inflamatórias crônicas, malignidade e uso de terapia imunossupressora, bem como pacientes com dados incompletos.

Este estudo incluiu pacientes submetidos à esternotomia mediana sob anestesia geral pela mesma equipe cirúrgica. Todos os pacientes receberam terapia anticoagulante padrão com heparina não fracionada intravenosa. Canulação aórtica padrão e venosa de duplo estágio foi aplicada aos pacientes que atingiram o valor efetivo de coagulação ativada após heparinização sistêmica. A cirurgia eletiva isolada de revascularização do miocárdio foi realizada induzindo parada cardíaca com cardioplegia sanguínea fria hipercalêmica anterógrada e CEC. Durante o procedimento cirúrgico, os pacientes foram resfriados a 32-33 ⁰C. A cardioplegia sanguínea fria foi administrada antegradamente a cada 20 minutos. A aplicação tópica de solução salina isotônica fria também foi utilizada nos pacientes nesse período. A artéria mamária interna esquerda foi utilizada como enxerto arterial e a veia safena magna como enxerto venoso. Após a remoção do pinçamento cruzado e o coração começar a bater, foram realizadas anastomoses distais sob o pinçamento cruzado e anastomoses proximais sob o pinçamento lateral em todos os pacientes.

Os pacientes foram divididos em dois grupos com base na presença de mortalidade operatória, que incluía qualquer morte, independentemente da causa, ocorrida dentro de dias de internação após a cirurgia. As causas de morte, que incluíram falência de múltiplos órgãos, disfunção neurológica permanente (acidente vascular cerebral/coma), insuficiência circulatória e choque tóxico infeccioso, foram determinadas com base na documentação da condição clínica em prontuários médicos.

### Análise estatística

A análise estatística foi realizada utilizando SPSS 23.0 (SPSS Company, Chicago, IL) para Windows. As variáveis contínuas foram descritas por meio de mediana e intervalo interquartil devido à falta de distribuição normal, testadas pelo teste de Shapiro-Wilk. Os testes U de Mann-Whitney foram utilizados para comparações entre os grupos de sobrevivência e não sobrevivência. Os testes qui-quadrado ou exato de Fisher foram utilizados para comparar os dois grupos em variáveis categóricas, que foram então resumidas por meio de contagens e porcentagens. A área sob a curva (AUC) da curva característica de operação do receptor (ROC) foi calculada para determinar o valor de corte ideal de SII, NLR e PLR para prever a mortalidade. Análises de regressão logística univariada e multivariada foram realizadas para identificar preditores independentes de ocorrência de mortalidade. Para análise secundária, para comparar os grupos de acordo com os valores de corte do SII, foi utilizado o teste U de Mann-Whitney. Um valor de p <0,05 foi considerado significativo.

## Resultados

Nos pacientes incluídos no estudo, um total de 78 pacientes desenvolveram mortalidade hospitalar. As características demográficas, perioperatórias e a análise laboratorial pré-operatória são apresentadas na Tabela 1 .

**Tabela 1:** Comparações demográficas e análises laboratoriais de rotina entre sobreviventes e não sobreviventes

Características	Sobreviventes (n=402)	Não sobreviventes (n=78)	Valor-p
Idade (anos)	63 (55-68)	67 (60-73)	<0,001
Gênero F/M, n (%)	79 (19,7)/323 (80,3)	28 (37,5)/50 (62,5)	0,002
Permanência Hospitalar (Dia)	14,0 (10,0-17,5)	9,0 (4,5-15,0)	<0,001
Permanência na Unidade de Terapia Intensiva (Dia)	4,0 (3,0-5,0)	3,0 (1,0-6,0)	<0,001
Permanência pós-operatória (dia)	7,0 (6,0-10,0)	2,0 (0,0-6,5)	<0,001
Duração do desvio cardiopulmonar (mín.)	100 (85-145)	105 (80-140)	0,637
Duração do grampo cruzado (min.)	50 (45-65)	55 (45-80)	0,328
**Parâmetros laboratoriais**			
Hemoglobina, g/dL	14h30 (13h00-15h40)	13,45(12,47-14,62)	0,005
WBC, 109/L	8,93 (7,15-11,09)	8,63 (7,06-11,51)	0,907
Neutrófilo, 109/L	5,61 (4,40-6,96)	6,28 (5,26-7,79)	0,069
Linfócito, 109/L	2,11 (1,62-2,74)	1,83 (1,28-2,37)	0,001
Proporção de linfócitos neutrófilos	2,62 (1,95-3,51)	3,50 (2,54-5,00)	<0,001
Plaquetas, 109/L	251,50 (215,00-292,50)	261,00 (235,00-299,25)	0,058
Proporção de linfócitos plaquetários	119,56 (90,03-156,18)	158,43 (112,90-210,53)	<0,001
Índice de Imuno-Inflamação Sistêmica (x109/L)	654,25 (465,49-936,46)	948,33(642,60-1355,60)	<0,001
Ureia, mg/dL	17h85 (13h97-22h30)	19h50 (14,67-24,82)	0,105
SCr, mg/dL	0,92 (0,80-1,10)	1,00 (0,82-1,27)	0,042
eGFR	84,49 (68,20-96,12)	77,93 (55,83-90,63)	<0,001
AST (U/L)	21,9 (17,65-32,07)	23h00 (18h00-34h00)	0,493
ALT (U/L)	21h00 (15h00-28h00)	19,3 (13,60-28,00)	0,244
Glicose, mg/dL	127 (104-177)	136 (112-195)	0,082
Triglicerídeo, mg/dL	178,00(164,00-213,00)	134,50 (107,75-171,25)	0,002
Colesterol total, mg/dL	186,00 (157,00-224,70)	175,00 (158,25-191,00)	0,024
Colesterol HDL, mg/dL	38,05 (35,07-43,92)	40,55 (34,75-45,00)	0,141
Colesterol LDL, mg/dL	105,00 (96,17-127,65)	103,74(86,55-121,72)	0,224
**Comorbidades, n (%)**			
Hipertensão	128 (34,1)	30 (53,6)	0,007
Doença renal crônica	9 (2,4)	6 (10,7)	0,007
Hiperlipidemia	6 (1,6)	1 (1,8)	0,999
Diabetes Mellitus	107 (28,5)	20 (39,7)	0,275
Doença pulmonar obstrutiva crônica	14 (3,7)	4 (7,1)	0,272
Tabagismo	6 (1,6)	1 (1,8)	0,999

p: Diferenças estatísticas entre sobreviventes e não sobreviventes. Os dados foram expressos como mediana e intervalo interquartil; %50 (%25-%75). As variáveis categóricas são expressas em porcentagens.

Na análise univariada, as seguintes variáveis pré-operatórias foram consideradas preditoras significativas de mortalidade: sexo, idade, SII, NLR, PLR, hemoglobina, SCr, eGFR, triglicerídeos, C-total, hipertensão e doença renal crônica ( Tabela 2 ). Uma análise multivariada dos mesmos parâmetros revelou que sexo, idade e SII, SCr, triglicerídeos e hipertensão permaneceram preditores independentes de mortalidade hospitalar ( Tabela 2 ).

**Tabela 2:** Análise de regressão logística univariada e multivariada dos fatores de risco pré-operatórios para predição de mortalidade

Variáveis	Análise Univariada				Análise multivariada			
	OR	IC 95%		Valor-p	OR	IC 95%		Valor-p
		Inferior	Superior			Inferior	Superior	
Gênero	0,438	0,259	0,740	0,002	2.129	1.204	3.766	0,009
Idade	1,043	1,016	1,071	0,002	1,029	1,001	1,058	0,044
SII	1,002	1,001	1,003	<0,001	1,003	1,001	1,005	<0,001
NLR	1,176	1,084	1,275	<0,001				
PLR	1,005	1,002	1,008	<0,001				
Hemoglobina	0,853	0,753	0,965	0,012				
SCr	1,356	1,069	1,721	0,012	1,523	1,173	1,977	0,002
eGFR	0,982	0,972	0,992	0,001				
Triglicerídeo	0,996	0,993	0,999	0,021	0,995	0,991	0,998	0,005
Colesterol total	0,889	0,886	0,892	0,035				
Hipertensão	0,491	0,301	0,801	0,004	1,878	1,106	3,189	0,020
Doença renal crônica	0,285	0,107	0,761	0,012				

IC: intervalo de confiança; RNL: razão de linfócitos neutrófilos; RPL: proporção de linfócitos plaquetários; SII: índice de imuno-inflamação sistêmica.

**Figura 1:**
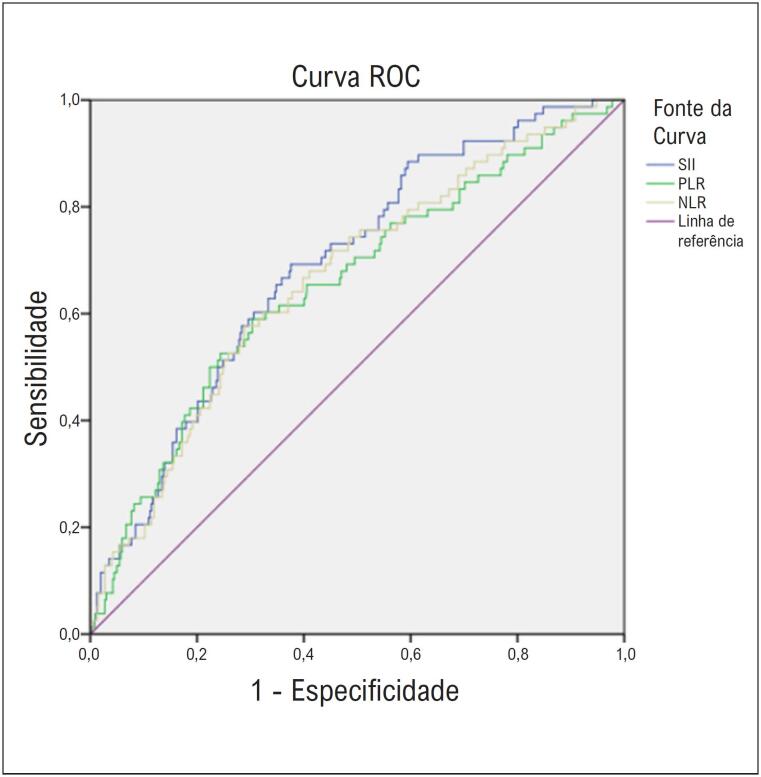
Análise da curva operacional do receptor de NLR, PLR e SII.

Pela análise das características operacionais do receptor, NLR, PLR e SII previram mortalidade em pacientes; a área sob a curva de 0,664 (IC 95% 0,599-0,729); 0,655 (IC 95% 0,587-0,723) e 0,690 (IC 95% 0,630-0,751), respectivamente ( Tabela 3 , Figura 1 ). Os valores de corte para NLR, PLR e SII para prever mortalidade hospitalar foram 3,31 (58% de sensibilidade, 71% de especificidade), 132,76 (65% de sensibilidade, 60% de especificidade) e 811,93 (65% de sensibilidade, 65% de especificidade), respectivamente ( Tabela 3 ).

**Tabela 3:** Valores de corte apropriados de NLR, PLR e SII

	AUC	Valor-p	% 95 IC		Valor de corte	Sensibilidade	Especificidade
			Inferior	Superior			
NLR	0,664	<0,001	0,599	0,729	3.31	0,577	0,711
PLR	0,655	<0,001	0,587	0,723	132,76	0,654	0,595
SII	0,690	<0,001	0,630	0,751	811.93	0,654	0,652

AUC: área sob a Curva; IC: intervalo de confiança; RNL: razão de linfócitos neutrófilos; RPL: proporção de linfócitos plaquetários; SII: Índice de Imuno-inflamação Sistêmica.

Em relação aos desfechos secundários, o tempo de internação hospitalar e pós-operatória foi significativamente maior para pacientes com níveis elevados de SII. No entanto, os tempos de permanência na unidade de terapia intensiva foram semelhantes entre os grupos ( Tabela 4 ). A Figura Central destaca os principais resultados do estudo.

**Tabela 4:** Resultados pós-operatórios para grupos SII

Características	SII ≥ 811,93 (n=173)	SII < 811,93 (n=307)	Valor-p
Permanência Hospitalar (Dia)	14,0 (10,0-17,0)	11,0 (7,0-17,0)	0,001
Permanência pós-operatória (dia)	7,0 (6,0-10,0)	2,0 (5,0-7,75)	0,022
Permanência na Unidade de Terapia Intensiva (Dia)	4,0 (3,0-5,0)	3,0 (2,0-5,0)	0,114

p: Diferenças estatísticas entre grupos. Os dados foram expressos como mediana e intervalo interquartil; %50 (%25-%75).

## Discussão

Na análise univariada, o presente estudo demonstrou que SII, hemoglobina, NLR, PLR, SCr, eGFR, triglicerídeos e Total-C poderiam ser usados como parâmetros de rotina para prever mortalidade hospitalar em pacientes submetidos à cirurgia cardíaca por CEC, juntamente com outros parâmetros clínicos e demográficos (idade, sexo, doença renal crônica e hipertensão). No entanto, o SII surgiu como parâmetro hematológico, e a SCr e os triglicerídeos como parâmetros bioquímicos, para prever de forma independente a mortalidade hospitalar após cirurgia de CEC.

Por diversos motivos, como contato do sangue com a superfície extracorpórea, trauma cirúrgico, endotoxemia e lesão de isquemia-reperfusão, a CEC resulta na liberação de citocinas pró-inflamatórias, que podem se transformar em cascata, levando a uma grave resposta imunoinflamatória no organismo. Acredita-se que a principal causa de morbidade e mortalidade pós-operatória seja a resposta do paciente.^1 , 15^ Como o desenvolvimento de estratégias preventivas nesta área requer a identificação de fatores de risco, principalmente durante o período pré-operatório, muitos estudos têm se concentrado nessa direção.

Os índices, calculados com fórmulas matemáticas simples, entre células de um hemograma completo, que muitas vezes é realizado rotineiramente, são considerados evidências valiosas para obter mais informações sobre a inflamação sistêmica. No entanto, os parâmetros leucocitários individuais são suscetíveis a alterações por condições externas (desidratação, hemodiluição, etc.), e os índices do tipo NLR são relativamente mais estáveis.^16^ Além disso, como esses índices permitem a avaliação conjunta de diferentes vias (imunes e inflamatórias), eles revelam resultados mais significativos do que sozinhos.^17 - 19^ A previsão de risco pela NLR e PLR tem sido estudada há muitos anos em muitos campos da medicina.^20 - 22^ Além disso, especialmente nos últimos anos, tanto a PLR quanto a NLR foram identificadas como marcadores significativos. para resultados pós-operatórios de cirurgia.^17 , 18 , 23 , 24^ Em uma meta-análise de 3.108 pacientes, Tan et al. mostraram que altos níveis de NLR pré-operatórios estavam associados à mortalidade e morbidade por todas as causas.^23^ Outro estudo realizado por Parlar e Şaşkın relatou que PLR e NLR, medidos tanto no pré quanto no pós-operatório, estavam associados à lesão renal aguda (LRA) pós-operatória, e eles relataram que os valores medidos no pós-operatório foram mais preditivos.^25^ Em contrapartida, Navani et al. e He et al., que investigaram a relação entre RPL e o desenvolvimento de fibrilação atrial pós-operatória e RNL e LRA, respectivamente, não conseguiram mostrar uma relação significativa.^26 , 27^ Esses resultados conflitantes podem estar associados a diferenças na metodologia estatística e na população do estudo. Outra razão para a não padronização das medidas de resultados utilizadas também pode ser considerada.^28^

Até onde sabemos, esta é a primeira vez na literatura que um novo índice, SII, usado para desfechos clínicos para diversos tipos de câncer, avaliou a mortalidade hospitalar em pacientes que usaram CEC além de RNL e RPL. O trabalho de Selcuk et al. é o mais semelhante ao presente estudo.^29^ No entanto, diferentemente do nosso estudo, que analisou a mortalidade intra-hospitalar, eles examinaram as relações entre SII, NLR e PLR pré-operatórios e o desenvolvimento de fibrilação atrial pós-operatória (FAPO). Eles descobriram que, semelhantemente ao presente estudo, todos os três índices foram significativos nas análises univariadas, mas apenas o SII poderia ser considerado um fator de risco independente nas análises multivariadas. No entanto, mostraram maior valor preditivo para SII (AUC: 0,7107) para FAPO em comparação com NLR e PLR (AUC: 0,6740 e 0,6426, respectivamente). Essa diferença pode ser explicada pelo fato de que os fatores que podem causar a mortalidade e sua patogênese estão distribuídos por uma faixa mais ampla. Isto também foi consistente com o argumento de Navani et al. em relação à PLR inalterada em pacientes submetidos à cirurgia cardíaca, uma vez que o efeito das plaquetas na patogênese da FAPO não é pronunciado.^26^

Porém, embora não tenham utilizado o SII diretamente, há estudos na literatura que utilizam a relação neutrófilos/linfócitos*plaquetas (NLPR), que é calculada de forma diferente.^19 , 30 , 31^ Koo et al. sugeriram que um aumento da relação NLPR pré-operatória estava associado a uma sobrevida pobre em longo prazo e que a NLPR pré-operatória pode ser um marcador preditivo independente superior de sobrevida em cinco anos do que a NLR pré-operatória e a contagem de plaquetas.^19^ Pelo contrário, Abanoz e Engin não conseguiram mostrar uma relação entre o desenvolvimento de eventos adversos maiores, incluindo mortalidade hospitalar, após cirurgia de revascularização do miocárdio e NLPR pré-operatória. Entretanto, após pós-cardiotomia, mostraram que a NLPR é um fator de risco independente mais preditivo.^31^

No presente estudo, idade, sexo, triglicerídeos, níveis de SCr e presença de hipertensão também foram encontrados como fatores de risco independentes. Os resultados são geralmente compatíveis com estudos semelhantes sobre morbidade e mortalidade na literatura.^27 , 32 - 34^ A CrS, que é um parâmetro muito importante na avaliação das funções renais, foi associada a mau prognóstico em pacientes de cirurgia cardíaca.^35^ Doenças cardíacas e renais, tanto agudas quanto crônicas, interagem ao longo de muitas vias comuns, incluindo mecanismos inflamatórios e imunológicos.^36^ Portanto, a insuficiência renal aprofunda ainda mais a insuficiência cardíaca e contribui para um aumento na mortalidade dos pacientes. Além disso, descobriu-se que a hipertensão também está associada ao acidente vascular cerebral, que é uma causa comum de morte.^33^

No entanto, o presente estudo tem algumas limitações. Primeiro, este foi um estudo unicêntrico. Portanto, o efeito do manejo perioperatório e cirúrgico, bem como as características do paciente, poderiam potencialmente distorcer os resultados, e pode-se dizer que nosso estudo tem uma amostra relativamente pequena. Em segundo lugar, tem um desenho retrospectivo, que apresenta preconceitos. A principal força deste estudo, por outro lado, é que este é o primeiro estudo a descrever a utilidade do SII como um parâmetro pré-operatório associado ao risco para mortalidade intra-hospitalar após CEC. Embora não seja um teste diagnóstico, esse parâmetro de rotina é útil como uma ferramenta de fácil acesso para prever possíveis complicações após a CEC.

## Conclusão

Altos escores pré-operatórios do SII podem ser usados para determinação precoce de tratamentos apropriados, o que pode melhorar os resultados cirúrgicos de cirurgia cardíaca no futuro. Além disso, quando os pacientes foram reagrupados com base nos valores de corte do SII no presente estudo, observou-se que a permanência hospitalar e o tempo de internação pós-operatória aumentaram significativamente no grupo com SII alto. Este estudo também ajudará a fornecer benefícios econômicos, pois esses resultados podem estar associados ao aumento dos custos de atendimento ao paciente. Acreditamos que nosso estudo inspirará mais pesquisas em larga escala sobre os efeitos adversos pós-operatórios.
