# Supplementary material for: O Índice de Imuno Inflamação Sistêmica Prevê Mortalidade Hospitalar em Pacientes Submetidos à Cirurgia Cardíaca com Circulação Extracorpórea
Source: Arq Bras Cardiol. 2024 Mar 28;121(4):e20230245. [Article in Portuguese] doi: 10.36660/abc.20230245 (PMC12092031; doi:10.36660/abc.20230245)
Supplement: Translation

# Translation: The Systemic Immune-Inflammation Index Predicts In-Hospital Mortality in Patients Who Underwent On-Pump Cardiac Surgery

**DOI:** 10.36660/abc.20230245i

**Authors:** İnayet Güntürk, Rifat Ozmen, Okan Ozocak, Ertuğrul Emre Güntürk, Fatma Dagli, Cevat Yazici

**Affiliations:** 1 Nigde Turkey Nigde Omer Halisdemir University, Faculty of Health Sciences, Midwifery, Nigde – Turkey; 2 Kayseri Turkey Erciyes University, Faculty of Medicine, Cardiovasculary Surgery, Kayseri – Turkey; 3 Istanbul Turkey Acibadem Hospitals Group – Cardiology, Istanbul – Turkey; 4 Kayseri Turkey Cetin Sen Science and Art Center – Chemistry, Kayseri – Turkey; 5 Kayseri Turkey Erciyes University Faculty of Medicine, Clinical Biochemistry, Kayseri – Turkey

**Keywords:** Extracorporeal Circulation, Inflammation, Hospital Mortality, Thoracic Surgery

## Abstract

**Background:**

Systemic immune-inflammation index (SII), a new inflammatory index calculated using platelet, neutrophil, and lymphocyte counts, has been demonstrated to be an independent risk factor for the identification of high-risk coronary artery disease in patients undergoing percutaneous coronary intervention and cardiovascular surgery with cardiopulmonary bypass (CPB). The relationship between SII and CPB-related mortality rates remains unclear.

**Objective:**

This research was designed to investigate the use of SII to predict in-hospital mortality in patients undergoing cardiac surgery with CPB.

**Methods:**

Four hundred eighty patients who underwent a cardiac procedure involving CPB over 3 years, were obtained from the hospital's database. The demographic data, comorbidities, hematological and biochemical profiles, and operative data of the groups were compared. Multiple logistic regression analyses were done to determine independent predictors of mortality. Prognostic factors were assessed by multivariate analysis, and the predictive values of SII, neutrophil-lymphocyte ratio (NLR), and platelet-lymphocyte ratio (PLR) for mortality were compared. A p-value <0.05 was considered significant.

**Results:**

Of 480 patients, 78 developed in-hospital mortality after cardiac surgery. SII was an independent predictor of in-hospital mortality (Odds ratio: 1.003, 95% confidence interval: 1.001-1.005, p<0.001). The cut-off value of the SII was >811.93 with 65% sensitivity and 65% specificity (area under the curve: 0.690). The predictive values of SII, PLR, and NLR were close to each other.

**Conclusion:**

High preoperative SII scores can be used for early determination of appropriate treatments, which may improve surgical outcomes of cardiac surgery in the future.

## Introduction

Extracorporeal circulation techniques, especially cardiopulmonary bypass (CPB) during cardiac surgery, provide blood flow and oxygen to tissues and organs.^1^CPB has become the standard for many cardiac procedures, and its advancement is enabling advances in cardiovascular surgery. The procedure is considered to be relatively safe.^2^CPB, on the other hand, is well known for initiating an inflammatory reaction cascade, and this inflammatory response has serious clinical consequences.^2 - 4^As a result, investigating the role of inflammatory changes in patient prognosis has become a high-priority target for the development of both therapeutic and preventive strategies.^5^For this purpose, many mediators such as pro-inflammatory cytokine levels such as IL-6, TNF, coagulation/fibrinolytic system, and complement activation markers have been investigated in relation to the CPB-related inflammatory response.^5 , 6^

However, when factors such as ease of measurement and interpretation, as well as low cost, are considered, such blood count parameters continue to be widely used. Although an increase in the number of white blood cells is accepted as a general marker for the inflammatory response triggered by various stimulants, its predictive value is insufficient.^7^Therefore, there is a recent emphasis on hematologic indices, such as neutrophil-lymphocyte ratio (NLR) and platelet-lymphocyte ratio (PLR), as markers of perioperative inflammation, considering the proposition of postoperative complications.^8^

The systemic immune-inflammation index (SII), a new inflammatory index calculated using platelet, neutrophil, and lymphocyte counts, has been shown to be a strong prognostic marker in a variety of cancers.^9 , 10^In addition, SII has been shown to be an independent risk factor for the identification of high-risk coronary artery disease in patients undergoing percutaneous coronary intervention, postoperative atrial fibrillation after coronary artery bypass grafting, a prognostic factor for infective endocarditis, and presence of isolated coronary artery ectasia.^11 - 14^However, the relationship between SII and CPB-related complications, especially in terms of in-hospital mortality rate, remains unclear.

In light of this, the current study sought to elucidate the independent association between SII and the occurrence of short-term mortality in patients who underwent on-pump cardiac surgery in order to provide a predictive marker for therapeutic interventions as early as possible.

## Methods

The study was conducted and approved by the Local Ethics Committee (Approval number: 2021/608). Due to the retrospective nature of the study, individual informed written consent was waived.

Patients who underwent a cardiac procedure involving CPB over 3 years (from 2018 to 2021) were collected. There was no operation-based selection used. Preoperative data for 480 adult patients was obtained from the hospital's database. The following data was collected for all the subjects: demographics (age, gender, smoking); the presence of comorbidities (chronic obstructive pulmonary disease, arterial hypertension, dyslipidemia, diabetes on medication, and chronic renal failure); preoperative laboratory results [complete blood count (CBC), blood urea nitrogen (BUN), serum creatinine (SCr), AST, ALT, total cholesterol (Total-C), HDL-C, LDL-C, glukoz, etc.]; type of surgical operation, CPB and aortic cross-clamp duration. The SII was defined as platelet count x neutrophil/lymphocyte count. Patients undergoing preoperative extracorporeal life support, active infection, chronic inflammatory conditions, malignancy, and the use of immunosuppressive therapy, as well as patients with incomplete data, were excluded.

This study included patients who underwent median sternotomy under general anesthesia by the same surgical team. All patients received standard anticoagulant therapy with intravenous unfractionated heparin. Standard aortic and dual-stage venous cannulation was applied to the patients who reached the effective activated coagulation value after systemic heparinization. Isolated elective CABG surgery was carried out by inducing cardiac arrest with antegrade hyperkalemic cold blood cardioplegia and CPB. During the surgical procedure, patients were cooled to 32-33 ⁰C. Cold blood cardioplegia was administered antegradely every 20 minutes. The topical cold isotonic saline application was also used on the patients during this period. The left internal mammary artery was used as an arterial graft, and the great saphenous vein was used as a venous graft. After the cross-clamp was removed and the heart started to beat, distal anastomoses were performed under the cross-clamp, and proximal anastomoses were performed under the side-clamp in all patients.

The patients were divided into two groups based on the presence of operative mortality, which included any death, regardless of cause, occurring within days of hospital stay after surgery. The causes of death, which included multiple organ failure, permanent neurologic dysfunction (stroke/coma), circulatory failure, and infectious toxic shock, were determined based on the clinical condition documentation in medical records.

### Statistical analysis

Statistical analysis was performed using SPSS 23.0 (SPSS Company, Chicago, IL) for Windows. Continuous variables were described using median and interquartile range due to their lack of normal distribution, tested using the Shapiro-Wilk test. Mann-Whitney U tests were used for comparisons between the survival and non-survival groups. The Chi-square or Fisher's exact tests were used to compare both groups on categorical variables, which were then summarized using counts and percentages. The area under the curve (AUC) of the receiver operating characteristic (ROC) curve was calculated to determine the optimal cut-off value of SII, NLR, and PLR to predict mortality. Univariate and multivariate logistic regression analyses were performed to identify independent predictors of the occurrence of mortality. For secondary analysis, to compare groups according to cut-off values of SII, the Mann-Whitney U test was used. A p-value <0.05 was considered significant.

## Results

In patients included in the study, a total of 78 patients developed in-hospital mortality. The demographic and perioperative characteristics and preoperative laboratory analysis are presented in Table 1 .

**Table 1:** Comparisons of demographics and routine laboratory analysis between survivors and non-survivor

Characteristics	Survivors (n=402)	Non-survivors (n=78)	p Value
Age (year)	63 (55-68)	67 (60-73)	<0.001
Gender F/M, n (%)	79 (19.7)/323 (80.3)	28 (37.5)/50 (62.5)	0.002
Hospital Stay (Day)	14.0 (10.0-17.5)	9.0 (4.5-15.0)	<0.001
Intensive Care Unit Stay (Day)	4.0 (3.0-5.0)	3.0 (1.0-6.0)	<0.001
Postoperative Stay (Day)	7.0 (6.0-10.0)	2.0 (0.0-6.5)	<0.001
Cardiopulmonary Bypass Duration (Min.)	100 (85-145)	105 (80-140)	0.637
Cross-Clamp Duration (Min.)	50 (45-65)	55 (45-80)	0.328
**Laboratory parameters**			
Haemoglobin, g/dL	14.30 (13.00-15.40)	13.45(12.47-14.62)	0.005
WBC, 10^9^/L	8.93 (7.15-11.09)	8.63 (7.06-11.51)	0.907
Neutrophil, 10^9^/L	5.61 (4.40-6.96)	6.28 (5.26-7.79)	0.069
Lymphocyte, 10^9^/L	2.11 (1.62-2.74)	1.83 (1.28-2.37)	0.001
Neutrophil Lymphocyte Ratio	2.62 (1.95-3.51)	3.50 (2.54-5.00)	<0.001
Platelet, 10^9^/L	251.50 (215.00-292.50)	261.00 (235.00-299.25)	0.058
Platelet Lymphocyte Ratio	119.56 (90.03-156.18)	158.43 (112.90-210.53)	<0.001
Systemic Immune-Inflammation Index (x10^9^/L)	654.25 (465.49-936.46)	948.33(642.60-1355.60)	<0.001
Urea, mg/dL	17.85 (13.97-22.30)	19.50 (14.67-24.82)	0.105
SCr, mg/dL	0.92 (0.80-1.10)	1.00 (0.82-1.27)	0.042
eGFR	84.49 (68.20-96.12)	77.93 (55.83-90.63)	<0.001
AST (U/L)	21.9 (17.65-32.07)	23.0 (18.00-34.00)	0.493
ALT (U/L)	21.0 (15.00-28.00)	19.3 (13.60-28.00)	0.244
Glucose, mg/dL	127 (104-177)	136 (112-195)	0.082
Triglyceride, mg/dL	178.00(164.00-213.00)	134.50 (107.75-171.25)	0.002
Total cholesterol, mg/dL	186.00 (157.00-224.70)	175.00 (158.25-191.00)	0.024
HDL-cholesterol, mg/dL	38.05 (35.07-43.92)	40.55 (34.75-45.00)	0.141
LDL-cholesterol, mg/dL	105.00 (96.17-127.65)	103.74(86.55-121.72)	0.224
**Comorbidities, n (%)**			
Hypertension	128 (34.1)	30 (53.6)	0.007
Chronic renal disease	9 (2.4)	6 (10.7)	0.007
Hyperlipidemia	6 (1.6)	1 (1.8)	0.999
Diabetes Mellitus	107 (28.5)	20 (39.7)	0.275
Chronic Obstructive Pulmonary Disease	14 (3.7)	4 (7.1)	0.272
Smoking	6 (1.6)	1 (1.8)	0.999

p: Statistically differences between survivors and non-survivors. The data were expressed as median and interquartile range; %50 (%25- %75). Categorical variables are expressed as percentages.

On univariate analysis, the following preoperative variables were found to be significant predictors of mortality: gender, age, SII, NLR, PLR, hemoglobin, SCr, eGFR, triglyceride, Total-C, hypertension, and chronic renal disease ( Table 2 ). A multivariate analysis of the same parameters revealed that gender, age, and SII, SCr, triglyceride, and hypertension remained independent predictors of in-hospital mortality ( Table 2 ).

**Table 2:** Univariate and Multivariate Logistic Regression Analysis of the Preoperative Risk Factors for Predicting Mortality

Variables	Univariate Analysis				Multivariate Analysis			
	OR	95 % CI		p Value	OR	95 % CI		p Value
		Lower	Upper			Lower	Upper	
Gender	0.438	0.259	0.740	0.002	2.129	1.204	3.766	0.009
Age	1.043	1.016	1.071	0.002	1.029	1.001	1.058	0.044
SII	1.002	1.001	1.003	<0.001	1.003	1.001	1.005	<0.001
NLR	1.176	1.084	1.275	<0.001				
PLR	1.005	1.002	1.008	<0.001				
Hemoglobin	0.853	0.753	0.965	0.012				
SCr	1.356	1.069	1.721	0.012	1.523	1.173	1.977	0.002
eGFR	0.982	0.972	0.992	0.001				
Triglyceride	0.996	0.993	0.999	0.021	0.995	0.991	0.998	0.005
Total cholesterol	0.889	0.886	0.892	0.035				
Hypertension	0.491	0.301	0.801	0.004	1.878	1.106	3.189	0.020
Chronic renal disease	0.285	0.107	0.761	0.012				

CI: confidence interval; NLR: neutrophil lymphocyte ratio; PLR: platelet lymphocyte ratio; SII: Systemic Immune-Inflammation Index.

By receiver operating characteristic analysis, NLR, PLR, and SII predicted mortality in patients; the area under the curve of 0.664 (95% CI 0.599-0.729); 0.655 (95% CI 0.587-0.723), and 0.690 (95% CI 0.630-0.751), respectively ( Table 3 , Figure 1 ). The cut-off values for NLR, PLR, and SII for predicting in-hospital mortality were 3.31 (58% sensitivity, 71% specificity), 132.76 (65% sensitivity, 60% specificity), and 811.93 (65% sensitivity, 65% specificity), respectively ( Table 3 ).

**Table 3:** Appropriate Cut-off Values of NLR, PLR, and SII

	AUC	p Value	% 95 CI		Cut-off Value	Sensitivity	Specificity
			Lower	Upper			
NLR	0.664	<0.001	0.599	0.729	3.31	0.577	0.711
PLR	0.655	<0.001	0.587	0.723	132.76	0.654	0.595
SII	0.690	<0.001	0.630	0.751	811.93	0.654	0.652

AUC: area under the curve; CI: confidence interval; NLR: neutrophil lymphocyte ratio; PLR: platelet lymphocyte ratio; SII: Systemic Immune-Inflammation Index.

**Figure 1:** Receiver Operating Curve Analysis of NLR, PLR and SII .

Regarding secondary outcomes, the duration of hospital and postoperative stays was significantly longer for patients with high SII levels. However, the durations of intensive care unit stays were similar between groups ( Table 4 ). The central illustration highlights the main results of the study.

**Table 4:** Postoperative outcomes for SII groups

Characteristics	SII ≥ 811.93 (n=173)	SII < 811.93 (n=307)	p Value
Hospital Stay (Day)	14.0 (10.0-17.0)	11.0 (7.0-17.0)	0.001
Postoperative Stay (Day)	7.0 (6.0-10.0)	2.0 (5.0-7.75)	0.022
Intensive Care Unit Stay (Day)	4.0 (3.0-5.0)	3.0 (2.0-5.0)	0.114

p: Statistically differences between groups. The data were expressed as median and interquartile range; %50 (%25- %75).

## Discussion

On univariate analysis, the current study demonstrated that SII, hemoglobin, NLR, PLR, SCr, eGFR, triglyceride, and Total-C could be used as routine parameters for predicting in-hospital mortality in patients who underwent cardiac surgery by CPB, along with other clinical and demographic parameters (age, gender, chronic renal disease, and hypertension). However, SII emerged as a hematologic, and SCr and triglyceride as biochemical parameters, to independently predict in-hospital mortality after CPB surgery.

For many reasons, such as contact of blood with the extracorporeal surface, surgical trauma, endotoxemia, and ischemia-reperfusion injury, CPB results in the release of pro-inflammatory cytokines, which can be transformed into a cascade, leading to a serious immune-inflammatory response in the organism. The primary cause of postoperative morbidity and mortality is thought to be the patient's response.^1 , 15^Since the development of preventive strategies in this area requires the identification of risk factors, particularly during the preoperative period, many studies have focused on this direction.

Indices, calculated with simple mathematical formulas, between cells from a CBC, which is often routinely performed, are sought to be valuable evidence to gain further information on systemic inflammation. However, individual leukocyte parameters are susceptible to alteration by external conditions (dehydration, hemodilution, etc.), and NLR-like indices are relatively more stable.^16^Also, since these indices allow the evaluation of different pathways (immune and inflammatory) together, they reveal more meaningful results than alone.^17 - 19^Risk prediction by the NLR and PLR has been studied for many years in many fields of medicine.^20 - 22^Moreover, especially in recent years, both PLR and NLR have been identified as significant markers for postoperative surgery outcomes.^17 , 18 , 23 , 24^In a meta-analysis of 3108 patients, Tan et al. showed that high preoperative NLR levels were associated with all-cause mortality and morbidity.^23^Another study by Parlar and Şaşkın reported that PLR and NLR, measured both preoperatively and postoperatively, were associated with postoperative acute kidney injury (AKI), and they reported that the values measured in the postoperative period were more predictive.^25^In contrast, Navani et al. and He et al., who investigated the relationship between PLR and the development of postoperative atrial fibrillation and NLR and AKI, respectively, could not show a significant relationship.^26 , 27^These conflicting results may be associated with differences in statistical methodology and study population. Another reason for the non-standardization of the outcome measures used can also be considered.^28^

To the best of our knowledge, this is the first time in the literature that a new index, SII, which is used for clinical outcomes for several cancers, was evaluated in-hospital mortality in patients who used CPB in addition to NLR and PLR. The work of Selcuk et al. is the most similar to the current study.^29^However, unlike our study, which looked at in-hospital mortality, they examined the relationships between preoperative SII, NLR, and PLR and the development of postoperative atrial fibrillation (POAF). They found that similarly to the current study, all three indexes were significant in univariate analyses, but only SII could be considered an independent risk factor in multivariate analyses. However, they showed a higher predictive value for SII (AUC: 0.7107) for POAF compared to NLR and PLR (AUC: 0.6740 and 0.6426, respectively). This difference can be explained by the fact that the factors that may cause mortality and their pathogenesis are spread over a wider range. This was also consistent with the argument by Navani et al. regarding the unchanged PLR in patients undergoing cardiac surgery, as the effect of platelets on the pathogenesis of POAF is not pronounced.^26^

However, although they did not use the SII directly, there are studies in the literature that use the neutrophil/lymphocyte* platelet ratio (NLPR), which is calculated using a different way.^19 , 30 , 31^Koo et al. suggested that an increased preoperative NLPR ratio was associated with poor long-term survival and that preoperative NLPR may be a superior independent predictive marker of five-year survival than preoperative NLR and platelet counts.^19^On the contrary, Abanoz and Engin could not show a relationship between the development of major adverse events, including in-hospital mortality, after coronary artery bypass grafting and preoperative NLPR. However, after post-cardiotomy, they showed NLPR to be a more predictive independent risk factor.^31^

In the current study, age, gender, triglyceride, SCr levels, and the presence of hypertension were also found as independent risk factors. The results are generally compatible with similar studies on morbidity and mortality in the literature.^27 , 32 - 34^SCr, which is a very important parameter in the evaluation of renal functions, was associated with poor prognosis in cardiac surgery patients.^35^Cardiac and renal diseases, acutely as well as chronically, interact along many common pathways, including inflammatory and immunological mechanisms.^36^Therefore, renal failure further deepens cardiac failure and contributes to an increase in mortality in patients. Furthermore, hypertension was also found to be associated with stroke, which is a common cause of death.^33^

Nonetheless, the current study has some limitations. First, this was a single-center study. Therefore, the effect of perioperative and surgical management, as well as patient characteristics, could potentially skew the results, and it can be said that our study has a relatively small sample size. Second, it has a retrospective design, which has biases. The main strength of this study, on the other hand, is that this is the first study to describe the utility of SII as a preoperative risk-associated parameter for in-hospital mortality after CPB. Although not a diagnostic test, this routine parameter is useful as an easily accessible tool for predicting potential complications after CPB.

## Conclusion

High preoperative SII scores can be used for early determination of appropriate treatments, which may improve surgical outcomes of cardiac surgery in the future. In addition, when patients were regrouped based on SII cut-off values in the current study, it was observed that hospital stay and postoperative length of stay increased significantly in the SII high group. This study will also help provide economic benefits as these results can be associated with increased patient care costs. We believe that our study will inspire more large-scale research into postoperative adverse effects.
